# A set of dual promoter vectors for high throughput cloning, screening, and protein expression in eukaryotic and prokaryotic systems from a single plasmid

**DOI:** 10.1186/1472-6750-12-54

**Published:** 2012-08-23

**Authors:** Namita Sinah, Charlotte A Williams, Robert C Piper, S Brookhart Shields

**Affiliations:** 1 Department of Molecular Physiology and Biophysics, Roy J and Lucille A Carver College of Medicine, University of Iowa, Iowa City, IA, 52242, USA; 2 Department of Biology, Luther College, Decorah, IA, 52101, USA

## Abstract

**Background:**

The ability to produce the same recombinant protein in both prokaryotic and eukaryotic cells offers many experimental opportunities. However, the cloning of the same gene into multiple plasmids is required, which is time consuming, laborious and still may not produce soluble, stable protein in sufficient quantities. We have developed a set of expression vectors that allows for ligation-independent cloning and rapid functional screening for protein expression in both *E. coli* and *S. cerevisiae.*

**Results:**

A set of expression vectors was made that can express the same open reading frame in *E. coli* (via the T7 phage promoter) and in *S. cerevisiae* (via the *CUP1* or *MET25* promoter). These plasmids also contain the essential elements for replication and selection in both cell types and have several advantages: they allow for cloning of genes by homologous recombination in yeast, protein expression can be determined before plasmid isolation and sequencing, and a GST-fusion tag is added to aid in soluble expression and purification. We have also included a TEV recognition site that allows for the specific cleavage of the fusion proteins to yield native proteins.

**Conclusions:**

The dual promoter vectors can be used for rapid cloning, expression, and purification of target proteins from both prokaryotic and eukaryotic systems with the ability to study post-translation modifications.

## Background

Often times, the gathering of reagents to preform a specific experiment can be more difficult than the execution of the experiment itself. This can especially true of experiments that examine the molecular interactions between proteins, which require the expression and purification of recombinant protein(s) from both eukaryotic and prokaryotic systems. Both systems have unique advantages for recombinant protein expression: prokaryotic *E. coli* allow for large quantities of recombinant protein to be easily and rapidly expressed and *S. cerevisiae* allow for eukaryotic proteins to be expressed with native binding partners and for *in vivo* function assessment. However, for the same protein to be produced in both systems multiple expression plasmids are typically used since the elements needed for efficient replication, selection, mRNA transcription, and protein translation for the two systems are different. The construction and verification of these multiple plasmids is time consuming, laborious, and error prone. Additionally, plasmids must be shuttled between different host strains for the cloning and expression steps.

We set out to overcome these experimental hurdles by combining into a single vector all of the essential elements for recombinant protein expression in both bacteria and yeast in a one-step, ligation-independent process. The benefits of expression plasmids that work in both bacterial and other eukaryotic cells have been noted previously [1,2]. Additionally, these vector systems have also touted the benefit of one-step cloning. However, the use of existing vectors has been limited to recombinant protein expression in either mammalian or insect cells and leaves a gap in the available expression tools in yeast. The vectors described here have several advantages; (1) genes can be cloned into the vectors in a ligation-independent method by homologous recombination in yeast, (2) a screen for protein expression and functionality can be used to identify positive clones in yeast before isolation of the plasmid DNA, (3) a protein can be expressed in both bacterial and yeast from the same vector, (4) a GST-fusion tag, which can be removed by the treatment with TEV protease, is provided to enhance protein solublility and purification, and (5) the entire protocol is very time efficient taking less than a week (5 days, 6 hours of hands-on time) to complete. To our knowledge, these are the first examples of dual promoter vectors that allow for one-step cloning for both bacterial and yeast expression.

The pDEP vectors contain all of the fundamental features spanning plasmid replication to protein translation within both bacteria and yeast. The plasmids were named pC-DEP and pM-DEP for *D*ual *E*xpression *P*romoter (pDEP) and are otherwise identical except the open-reading frame of interest are under the control of either yeast *CUP1* (pC-DEP) or *MET25* (pM-DEP) promoters, respectively. The *CUP1* promoter drives intermediate levels of protein expression upon the addition cuprous ions to growth media [3]. The *MET25* promoter drives modest levels of expression in complete media, but is derepressed in the absence of methionine to drive high levels of protein expression [4]. For protein expression in bacteria, a T7 bacteriophage promoter and ribosome-binding site is located between the yeast promoter and the translation start codon. The vectors contain the *URA3* gene for growth selection in *ura3* mutant yeast grown on uracil deficient media. Also, the ampicillin resistance gene, β-lactamase (*bla*), is included for selection of bacteria grown on ampicillin-containing media. The yeast *CEN/ARS4* origin of replication and the bacterial pMB1 *ori* affords replication of the plasmid in the respective systems. An additional feature is the inclusion of a GST-fusion tag at either the N-terminus or C-terminus of the open-reading frame of interest to enhance protein solubility and protein purification. Combination of these features into a single plasmid streamlines the process of recombinant protein expression.

Despite of what appears to be a simple modular combination of the above-mentioned features, several unexpected difficulties had to be overcome in our studies to isolate a fully functional set of plasmids and procedures to enhance their performance. We demonstrated the utility of our system to rapidly clone, screen, express, and purify recombinant proteins by cloning 6 different ubiquitin-like (Ubl’s) proteins into the vectors.

## Results

### Design of pDEP vectors

**Figure 1:**
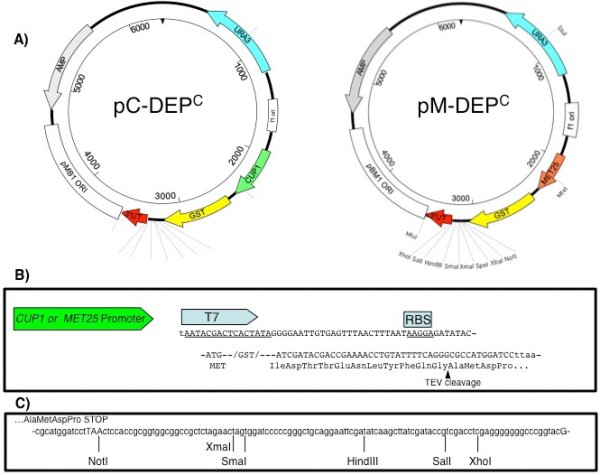
**Schematic representation of pDEPC vectors.** (**A**) Schematic of pC-DEP^C^ and pM-DEP^C^ vectors with the following features labeled: *URA3* ORF in blue, f1 ori in white, *CUP1* yeast promoters in green, *MET25* yeast promoters in orange, glutathione S-transferase gene (GST) in yellow, the *CYC1* yeast transcription terminator in red, the pBM1 origin of replication in white, and the *bla* gene conferring ampicillin resistance in grey. Unique restriction sites are labeled. (**B**) Nucleotide sequence of the translation initiation region and corresponding protein translation are shown. This region positions the prokaryotic T7 promoter and ribosomal binding site (RBS) after the eukaryotic CUP1 or MET25 promoters. A TEV protease cleavage site is located between the GST-tag and the insertion site for the ORF of interest. (**C**) Nucleotide sequence of the multiple cloning site (MCS).

The pDEP vectors were designed to include all of the essential elements for efficient replication, selection, mRNA transcription, and protein translation within both bacteria and yeast (Figure 1A and B). The yeast origin of replication chosen was the CEN6 and the ARS associated with Histone 4 (*CEN6/ARS4*). The *URA3* gene, which allows for growth on media lacking uracil, was included as an autotrophic selection marker in yeast. Protein expression in yeast is under the control of the copper inducible promoter from the *CUP1* gene or the methionine-regulated *MET25* promoter. The *CYC1* terminator was selected as the yeast translation terminator. The bacterial origin of replication, pMB1, and ampicillin resistance gene, β-lactamase (*bla*), were also selected for inclusion. The T7*lac* promoter was chosen to drive protein expression in bacteria and was placed directly after the yeast promoter and ~25 bp before a ribosome binding site (RBS). The T7*lac* promoter is a combination of the T7 bacteria phage promoter placed upstream of the lac operator (lacO) sequence, a binding site for the lac repressor. Protein expression is induced upon expression of T7 polymerase in DE3 lysogenized cells using IPTG and the lacO sequence functions to repress basal transcription of the gene of interest from the T7 polymerase. However, the final vectors did not contain the lacO sequence, see Discussion section for details.

Additionally, the coding sequence of Glutathione S-transferase (GST) protein was incorporated to allow for an in-frame fusion-tag. The pDEP^N^ and pDEP^C^ vectors allow for the gene of interest to be inserted at the N-terminus or C-terminus of the encoded GST fusion tag, respectively.

A tobacco etching virus (TEV) protease cleavage site is integrated between the GST-fusion tag and the coding sequence of the gene of interest in the pDEP^C^ vectors. When recombinant protein containing this site is treated with TEV protease, it is specifically cleaved at the TEV site to produce native protein. A multiple cloning site (MCS) was also included between the TEV cleavage site and the *CYC1* terminator to allow for linearization of the plasmid for the insertion of a gene of interest.

### Construction of pDEP vectors

The pDEP vectors were assembled by the homologous recombination of multiple PCR amplicons to a linearized target plasmid (as described in materials and methods). The target plasmid, pRS316, contained the yeast origin of replication (*CEN/ARS4*) and nutritional selection marker (*URA3*), and the pMB1 bacterial origin of replication and the ampicillin resistance gene, β-lactamase (*bla*). The PCR primers were designed to amplify the *CUP1* promoter, the T7*lac* promoter and ribosome binding sequence, the coding sequence for the GST-TEV fusion tag, and the *CYC1* terminator. The T7*lac* promoter was designed to have binding sites for both T7 RNA polymerase and LacI, which induces protein expression and prevents basal gene transcription in repressed bacteria, respectively. Engineered within the PCR primers were additional flanking sequences of ~50 bp, Additional file 1: Figure S1. The flanking sequences targeted the resulting linear amplicons for recombination with the intended neighboring sequences and the target plasmid via an “ends-out” double strand break repair mechanism. This was achieved by co-transformation of the PCR amplicons and linearized target plasmid into yeast. A fraction of the linearized plasmid simultaneously acquired the PCR amplicons and was recircularized. Recombinants that contain the recircularized plasmid encoding the *URA3* gene were selected for on media lacking uracil. Control transformations of linearized plasmid DNA alone or PCR amplicons alone did not yield a significant number of transformants (< 20) while the ‘complete’ transformation contained several hundred transformants (data not shown).

Each individual yeast transformant represented a single recombination event and contained a single plasmid. A plasmid that correctly recombined would contain all the necessary elements for replication, selection, mRNA transcription, and protein translation in yeast. Hence, the ability to produce recombinant GST protein was used as a read out for these correct recombination events. This was assessed by immunoblot analysis with α-GST antibodies of individual yeast transformants. When transformants were grown in liquid culture containing 100 μM CuCl_2,_ expression of GST protein was induced from the *CUP1* promoter. Surprisingly, after screening 50 yeast transformants, only one was found to produce high levels of GST protein. This plasmid was rescued from yeast and transformed into *E. coli*. Upon sequencing, it was found that this plasmid contained the T7 RNA polymerase-binding site but lacked the flanking sequence required for LacI binding. This suggested that by using screening in yeast, we were able to find a mutant variant capable of proper yeast expression. Upon introduction of the plasmid into BL21 (DE3) cells, the production of large amounts of GST-fusion protein was assessed using SDS-PAGE and staining with Coomassie Brilliant Blue. This initial plasmid then served as the basis for all of the modified pDEP vectors described below. For versatility, a multiple cloning site replaced the 3’ region of this initial plasmid to produce pC-DEP^C^.

The pM-DEP^C^ plasmid was constructed to have the *MET25* promoter in place of the *CUP1* promoter. The *MET25* promoter had the advantage that it is tightly regulated by the presence of methionine in growth media: strong protein expression is seen in the absences of methionine and is not detectable in the presence of 10 μg/ml methionine. A PCR amplicon was engineered to include the *MET25* promoter and ~50 bp of flanking sequence on each end. This was integrated in place of the *CUP1* promoter of pC-DEP^C^ to produce pM-DEP^C^, Figure 1.

**Figure 2:**
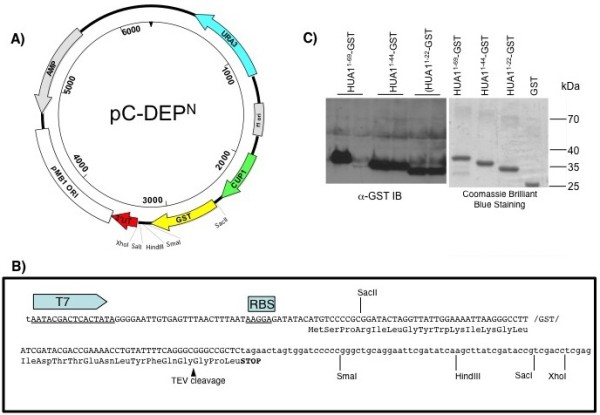
**Use of pC-DEP**^**N**^** plasmid for making untagged or N-terminal GST-fusion proteins.** Schematic of pC-DEP^N^ plasmid with the following features labeled: *URA3* ORF in blue, f1 ori in white, *CUP1* yeast promoters in green, glutathione S-transferase gene (GST) in yellow, the *CYC1* yeast transcription terminator in red, the pBM1 origin of replication in white, and the *bla* gene conferring ampicillin resistance in grey. Unique restriction sites are labeled. Nucleotide sequence of the translation initiation region and corresponding protein translation sequence of the pC-DEP^N^ plasmid. The unique SacII site allows for genes of interest to be recombined upstream of the GST coding sequence. Demonstration of the function of pC-DEP^N^ plasmid. Left panel: Western blot analysis using α-GST antibodies of yeast cultures expression the indicated residues of the Hua1 gene that were recombined into the pC-DEP^N^ plasmid. Right panel: Coomassie Brilliant Blue stained SDS-PAGE gel of the same GST-fusion proteins as in left panel purified from BL21 (DE3) cells using GSH-agarose.

In a similar manner, the pC-DEP^C^ plasmid was modified to produce pC-DEP^N^ plasmid. The pC-DEP^N^ plasmid allows for the expression of either native protein or with a C-terminal GST-fusion tag, Figure 2. pC-DEP^C^ differs from pC-DEP^N^ by the inclusion of SacII endonuclease site after the start codon of the GST coding sequences. (Note that the TEV cleavage site and MCS of the original pC-DEP^C^ plasmid remain intact). Once the pC-DEP^N^ is linearized at the SacII site, a gene of interest can be recombined upstream of the GST coding sequence. If the coding sequence of the gene of interest includes a stop codon, the recombinant protein produced within the native, untagged form. However, if it does not include a stop codon, the recombinant protein produced will include a C-terminal GST-fusion tag.

### Using the pDEP Vectors

**Figure 3:**
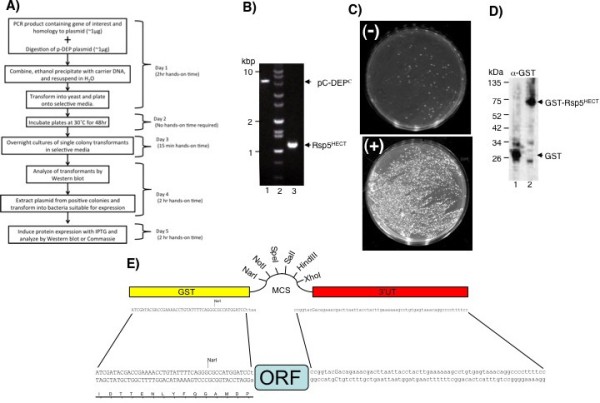
**Workflow of ligation-independent cloning into the pDEP vectors.** (**A**) Experimental steps, timeline, and hands-on time associated with each step of cloning a gene of interest into the pDEP vectors. From beginning to end, cloning take less than 5 days and ~6 hr of hands-on time. (**B**) Agarose separating gel visualized with ethidium bromide. Lane 1: digested vector pC-DEP, lane 2: DNA size marker, lane 3, PCR product of the HECT-domain of Rsp5 (Rsp5^HECT^, residues 446–809). (**C**) DNA fragments from Figure 3B was transformed into yeast and plated on selective media. Top plate (−), negative control containing only digested plasmid, and Bottom plate (+), successful recombination of PCR product and digested vector. (**D**) Western blot analysis for positive recombination events using α-GST antibodies. Lane 1, GST protein expression from pC-DEP^C^ not containing an ORF, and lane 2, expression of GST-Rsp5^HECT^ from recombination plasmid pC-DEP^C^- Rsp5^HECT^. (**E**) Schematic view of the plasmid insertion site and the PCR product designed to integrate into the pDEP plasmids via homologous recombination in yeast. The flanking ends can be generated as part of the PCR primers and will place the open reading frame of interest in frame with the 5’ GST open reading frame.

The workflow and time required for cloning a gene of interest into the pDEP vectors is outlined in Figure 3A. The first step involves preparing the target by digestion with endonucleases specific for the sites within the MCS of pC-DEP^C^ or pM-DEP^C^ or the SacII site within pC-DEP^N^. Simultaneously, a PCR amplicon containing the gene of interest and ~50 bp flanking sequences with homology to the target plasmid is made. The resulting DNA fragments are then prepared and co-transformed into yeast. The transformation mixture is plated on selective media lacking uracil and incubated for two days. On the third day, the individual yeast transformants are inoculated into selective media containing the proper protein induction condition: either 100 μM CuCl2 in the case of the *CUP1* promoter or absence of methionine in the case for the *MET25* promoter. The next day, day 5, individual transformants are then assayed for the production of the GST-fusion protein by immunoblot with α -GST antibodies. This screens for correct recombination events of the PCR amplicon with the target plasmid. The recircularized plasmid is then rescued from yeast transformants that show robust protein expression in the yeast cultures on the same day. Immediately this plasmid DNA can be transformed into a bacterial expression strain and the bacterial culture grown overnight. On day six, the bacterial culture in induced with the addition of IPTG to the culture media. Again, the culture can be assayed for recombinant protein expression by either the presence of the a band corresponding to the predicted molecular weight of the GST-fusion protein by SDS-PAGE gel stained with Coomassie Brilliant Blue or by probing with α-GST antibodies. Overall, the procedure takes a total of five days (6 hours hands-on time) to produce a plasmid that has a confirmed protein expression in both yeast and bacteria.

Figures 3B- 3D illustrate different steps in the workflow of cloning a gene of interest, in this case the HECT-domain of the ubiquitin ligase Rsp5, into the pC-DEP^C^ plasmid. Using the sequences shown in Figure 3E, PCR oligos were designed to amplify regions of the Rsp5 open reading frame and included ~50pb of homology to the pC-DEP^C^ vector. The resulting PCR product is shown in lane three of Figure 3B. The pC-DEP^C^ plasmid was linearized at restriction enzyme sites within the MCS, lane 1 in Figure 3B. The DNA fragments were then co-transformed into yeast and plated on selective media, Figure 3C, lower panel. We found that ample yeast transformants were typically obtained using ~1 μg each of linearized plasmid and PCR product. Figure 3C, upper panel displays the typical results from the control transformation of linearized plasmid alone. Comparison of the upper and lower panels of Figure 3C clearly illustrates the efficiency of recombination of the PCR amplicon with the target plasmid.

In order to screen for correct recombination events that produced a recircularized plasmid with the Rsp5 HECT-domain inserted, individual yeast transformants were analyzed by immunoblot with α-GST antibodies after growth in selective media containing CuCl_2._ These conditions induced expression of the GST-Rsp5^HECT^ fusion protein, which was under control of the *CUP1* promoter. An example of this assay is shown in Figure 3D. Lane one is recombinant GST protein (~26 kDa) produced from the pC-DEP^C^ vector without a gene insert and serves as a control. The dominant band in lane two of Figure 3D is at ~75 kDa, the predicted molecular weight for the GST-Rsp5^HECT^ fusion protein produced by the pC-DEP-Rsp5^HECT^ plasmid. In general, 5–10 yeast colonies were screened in this way for the presence of the GST-fusion protein. Typically, less than 10% failed to combine the gene of interest into the target plasmid (data not shown). These colonies either did not express any proteins detected with the α-GST antibodies or produced a band at ~26 kDa, representing GST alone. One major advantage of this workflow is that plasmids within individual transformants can be screened on the basis of their expression, ensuring that a plasmid can be identified that encodes a full-length protein and can be produced at desired levels.

### Protein expression and optimization

**Figure 4:**
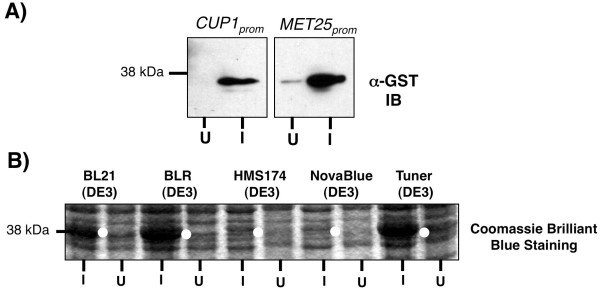
**Expression of protein in both bacteria and yeast from the pDEP**^**C**^** plasmids.** (**A**) Comparison protein expression levels from the CUP1 and MET25 promoters in yeast by Western blot analysis of yeast lysates using α-GST antibodies of GST-MVB12 fusion protein (38KDa). Left panel, protein expression from pC-DEP^C^-MVB12 plasmid under the control of the *CUP1* promoter was induced by the addition of copper chloride; and right panel is same protein expressed under control of the *MET25* promoter using pM-DEP^C^-MVB12 plasmid, induced by the absence of methionine. (**B**) Comparison of protein expression levels from the indicated *E. coli* strains transformed with the pC-DEP^C^-MVB12 plasmid. Lanes are labeled U for uninduced and I for induced with 0.2 mM IPTG for 2 hrs. Bacteria lysates were separated by SDS-PAGE gel stained by Coomassie Brilliant Blue. White circles in induced lanes indicate the GST-MVB12 fusion protein (38 kDa).

The pDEP set of vectors allows for control of protein production in yeast to be under the control of the *CUP1* promoter (pC-DEP^C^) or the *MET25* promoter (pM-DEP^C^). Comparison of protein expression from these two promoters is illustrated in Figure 4A. In this instance, the gene encoding the Class E Vps protein *MVB12* was cloned into both the pC-DEP^C^ and pM-DEP^C^ vectors. For the pC-DEP^C^-Mvb12 plasmid, the addition of CuCl_2_ (25–100 μM) to the culture media rapidly induces strong production of the GST-Mvb12 fusion protein at 38 kDa, right lane mark I in the left panel of Figure 4A. In addition, protein expression can be suppressed below the detectable range of the α-GST antibody by the addition of the copper chelating agent BCS to the culture media (left lane marked U, in left panel of Figure 4A). Low levels of expression is seen from the pC-DEP^C^ plasmid in media without either CuCl_2_ or BCS added, presumably due to residual amounts of cupric ions in the water used (data not show). The *MET25* promoter also yields robust and regulated protein expression. Protein expression from the *MET25* promoter is induced by growing cells in the absence of methionine and suppressed by the addition of 10 μg/ml methionine to the growth media (Figure 4A, left panel labeled I and U, respectively). In general the *CUP1* promoter produced tighter control over protein expression while the *MET25* promoter provided slightly stronger protein expression.

Bacterial protein expression from the pDEP plasmids is under control of the T7 RNA polymerase in DE3 lysogenized cells (λDE3). Robust protein expression was detected when 1 mM IPTG was added to culture media. However, the method used to transform λDE3 cells and, to a lesser extent, the specific strain of λDE3 cells was critical for expression in *E. coli*. We found that the pDEP plasmids could only be reliably transformed into λDE3 cells if the transformants were grown in media (or plated on media) containing a lower amount of amplicilin (25 μg/ml). Once transformants had grown overnight, the *E. coli* culture could be grown in media with up to 100 μg/ml ampicillin and robustly produced the desired recombinant protein (Additional file 2: Figure S2).

Several types of λDE3 bacterial strains were screened for the highest and most consistent level of recombinant protein production from the pC-DEP^C^-Mvb12 plasmid. In general, bacterial strains derived from the λDE3 lysogenized K-12 *E.coli* strains (HMS147 and NovaBlue, marked by white dots in Figure 4B) produced little or no detectable protein at the predicted molecular weight of 38 kDa. However, several λDE3 B strain derivatives including BL21, BLR, and Turner cells all worked very well, Figure 4B, marked by white dots. It was also found that comparable levels of recombinant protein were produced in bacteria when the same construct, GST-Mvb12, was contained in an *E. coli* expression plasmid pGEX-3X (data not shown).

**Figure 5:**
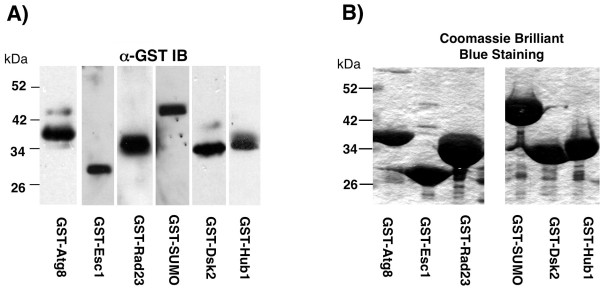
**Expression of multiple Ubl proteins from both bacteria and yeast in the pC-DEP**^**C**^** vector.** (**A**) Western blot analysis using α-GST antibodies of yeast lysates expressing the indicated GST-fusion proteins. The designated ubiquitin-like domains (Ubls) were recombined into the pC-DEP^C^ vector and induced with copper chloride. (**B**) Coomassie Brilliant Blue stained SDS-PAGE gel of GST-fusion proteins purified from bacteria (BL21(DE3)) transformed with the identical plasmids in Figure 5A.

To demonstrate the utility of the pC-DEP^C^ system, we generated a number of GST-fusion constructs in parallel (Figure 5). Several coding sequences for ubiquitin like (Ubl) proteins of yeast were amplified and recombined in yeast using linearized pC-DEP^C^. Recombinant plasmids were isolated from yeast and retransformed into yeast and bacteria (BL21(DE3)). Production of GST-Ubl proteins were induced with 100 μM CuCl_2_ in yeast and visualized by immunoblotting with α-GST antibodies. The GST-Ubl proteins produced in bacteria upon IPTG induction were purified using GSH-agarose resin and analyzed by SDS-PAGE and Coomassie Brilliant Blue staining. All of these constructs were made in the same time scale as the workflow described in Figure 3A, showing that this system is robust for making several functional constructs in parallel.

To test the functionality of the pC-DEP^N^ plasmid, which allows for C-terminal fusion of GST to proteins, several portions of the Hua1 gene were incorporated, Figure 2. After the pC-DEP^N^ plasmid was linearized with SacII endonuclease, it recombined PCR amplicons encoding the N-terminal 22, 44, or 69 residues of the yeast Hua1 protein. Two yeast colonies from each transformation were grown in CuCl_2_ overnight and analyzed for expression of the Hua1-GST fusion proteins by immunoblot analysis (Figure 2C). Plasmids were rescued from yeast and transformed into BL21 (DE3) cells. Recombinant proteins from bacterial cultures induced with IPTG were affinity purified over GSH-agarose and analyzed by SDS-PAGE and Coomassie Brilliant Blue staining (Figure 2C).

## Discussion

The pDEP vectors designed in this study allow for the rapid cloning of genes for expression of recombinant proteins in both prokaryotic and eukaryotic systems from a single vector. Genes of interest are cloned into the pDEP vectors in a ligation-independent manner by exploiting the ability of yeast to perform homologous recombination rapidly and efficiently. A gene of interest is targeted for integration into the pDEP vectors by the addition of specific homologous sequences, which are designed into the oligos for the gene of interest, by PCR amplification. This cloning procedure has several unique advantages over ligation-dependent subcloning methods. Firstly, traditional subcloning is a several step process requiring the gene of interest to be cloned from genomic DNA, put into a shuttle vector and subsequently transferred to an expression plasmid. Most often restriction enzymes are used and the efficiency of subcloning decreases with the increased size of the insert. Each step in this process has the potential for error and failure and requires sequencing to confirm the construct. Furthermore, restriction endonucleases used for these procedures may not be compatible with the gene of interest and render it useless for recombinant protein expression. Additionally, several plasmids are currently needed in order to express proteins in the two different systems, compounding the time and difficulty of the subcloning task. Finally, subcloning methods require the resulting plasmid to be transferred into an expression strain, which can lead to the discovery that the construct either does not produce protein in sufficient quantities or that it is not soluble. Many systems have been created (such as Invitrogens Gateway and TOPO cloning systems) to help overcome these hurdles, yet do not address all of these difficulties.

The pDEP vectors offer a cohesive and simple way to solve these problems. Cloning into the pDEP vectors is a one-step, ligation-independent process, streamlining the procedure greatly. A gene of interest can be directly amplified by PCR from genomic DNA using oligos designed with flanking sequences homologous to all of the pDEP vectors. This PCR amplicon can then be directly recombined into the target plasmid by transformation into yeast. This bypasses the problem of using restriction enzymes with the gene of interest. Homologous recombination is also not hampered by a large sized insert, as Gibson et. al. demonstrates the ability of *S. cerevisiae* to combine 25 DNA fragments ranging from 17 to 31kbp in size [5]. Similarly, homologous recombination provides the ability to produce chimeric proteins constructed from multiple coding sequences.

Moreover, the host strain for the recombination, yeast, is also able to serve as an expression strain when grown in the proper conditions to induce protein expression. This directly provides a critical readout of the quality and quantity of recombinant protein produced from the plasmid before it is isolated and sequenced. This is not possible with the commercially available systems. Lastly, the pDEP plasmid can then be transferred into a bacterial expression strain for recombinant protein expression in a prokaryotic system. This eliminates the need for additional subcloning and isolation steps and also the need for the sequencing of multiple constructs. From start to finish, the method takes roughly 5 days, including 6 hours of hands-on time, which is a significant timesaving over traditional subcloning.

It should be noted that there are commercially available technologies that would be compatible with the pDEP vectors. Although not tried here, it should be possible to obtain proper pDEP clones using ligation-independent cloning in *E.coli* using the In-Fusion enzyme (Clontech) and similar PCR products used for yeast recombination. However, this method would require the resulting plasmid be transferred to an expression strain, either *E.* coli or *S. cerevisiae.*

Having a bacterial/yeast expression system for recombinant protein expression offers other advantages beyond rapid vector construction and functional screening. Isolating the same protein from both yeast and bacteria allows for a more stringent evaluation of eukaryotic specific post-translational modifications by methods such as mass spectroscopy, SDS-PAGE mobility, and others. The same polypeptide could be expressed in the two systems and then the properties directly compared. For example, *Atg8p* is an integral component of the yeast autophagy system and is covalently attached to the membrane phospholipid phosphatidylethanolamine (PE) post-translationally. When produced in bacteria, the GST-ATG8 fusion protein appears at the predicted molecular weight of ~40 kDa, Figure 5B, left most lane. However, when produced in a eukaryotic system that includes the native conjugation machinery, additional bands are detected in for the GST-ATG8 fusion protein, Figure 5A, left most lane. A similar trend is seen for Dsk2p, a known target for ubiquitination, fifth lane in Figures 5A and 5B. The original pC-DEP^C^ vector would not be ideal for investigating all post-translational modifications in this way since the N-terminus is critical for directing proteins into specific cellular locations and is blocked by the GST-fusion tag. However, the pC-DEP^N^ plasmid allows for insertion of fragments upstream of the GST coding sequence, permitting the expression of either native, untagged protein or C-terminal GST-fusion tags. Additionally, pC-DEP^N^ plasmid would also enable recombination strategies where the entire GST ORF could be replaced with other protein fusion tags. Furthermore, the pDEP vectors could streamline surface mapping studies designed to pin-point domains that mediate protein:protein interaction. Using both bacterial and yeast expression systems could help differentiate between direct and indirect interactions.

During the construction of the initial pDEP vector, pC-DEP^C^, very few of the recombinants screened positive (1 in 50) for the production of GST protein in yeast. This was quite unexpected because there were a considerable number of transformants. However, sequencing of this plasmid showed that a coincidental recombination event had excised the lac operator (lacO) site of the T7*lac* promoter. The lac repressor-binding site had been positioned between the T7 promoter and the RBS and was initially included to allow for tighter control of protein production in DE3 lysogenized *E. coli*. When we repaired this error by site directed mutagenesis, the plasmid no longer produced GST to any detectable level in yeast (data not shown). It is not clear why this small, 5 bp region between the *CUP1* promoter’s transcriptional start site and the start of the open reading frame would be detrimental for expression in yeast. Our ability to screen through several yeast colonies at the initial stages of our study was critical to our ability to identify the rare plasmid variants that ultimately worked. This does explain why most of the initial yeast transformants did not express protein and underscores the advantage the pDEP system had in screening for plasmid variants with desired expression characteristics.

Another unexpected result in this study was the difficulty of reliably transforming pDEP plasmids into BL21(DE3) cells for bacterial expression. Transforming pC-DEP^C^ plasmid, or the parent yeast expression plasmid pRS316, fails to yield ampicillin-resistant cultures using standard transformation conditions (Additional file 2: Figure S2). Some benefit was observed upon adding 1% glucose to cells during their initial growth in ampicillin media (Additional file 2: Figure S2B). This was encouraging since it is known that glucose can repress basal levels of T7 RNA polymerase, which might create growth limiting products (RNA or protein) from pC-DEP^C^ or pRS316 plasmids, which themselves are high copy plasmids [6]. However, high levels of GST expression are not toxic for bacteria. We also observed transformation problems with yeast expression plasmids that contained no ORF downstream of the T7 promoter, suggesting that it is not production of the GST fusion proteins per se that were the source of the transformation problem. Rather it may be another RNA derived from the plasmid. In side by side comparisons, we found the transformation problem to be even worse in BL21 star (DE3) cells, which contain the *rne131* mutation that compromises the major enzyme responsible for mRNA degradation [7,8]. Efforts to limit T7 RNA polymerase activity by using BL21 (DE3) strains with pLysS (which encodes T7 lysosome and inhibits tT7 RNA polymerase thus lowering its basal activity) did not correct this defect. We also made a derivative of pC-DEP containing the ROP gene, which is known to diminish the copy number of plasmids that have the ColE1-related origin of replication (Additional file 2: Figure S2C). However, while this did reduce plasmid copy number as assessed by yield from plasmid preparation procedures, it did not restore transformation into BL21 under standard conditions (Additional file 2: Figure S2A and S2B). The easiest and most reliable remedy was simply to grow the initial cultures of BL21 (DE3) cells in media (LB) containing 25 μg/ml ampicillin after transformation rather than the 50–100 μg typically recommended for high copy plasmids (Additional file 2: Figure S2A). This level of ampicillin is high enough to select for the plasmid and consistently gave BL21 (DE3) transformants with all of our pDEP plasmids.

## Conclusion

In this study, a set of plasmids was developed for the expression of proteins in both prokaryotic and eukaryotic systems and named pDEP for *D*ual *E*xpression *P*romoter. Genes of interest are cloned into the vector in a ligation-independent manner, greatly simplifying and streamlining this procedure. The cloning procedure also allows for protein expression, solubility, and functionality to be screened before the plasmids are isolated and sequenced. Furthermore, the pDEP vectors were optimized to overcome several unforeseen experimental difficulties. These vectors advance the available tools to researchers working with both E*. coli* and *S. cerevisiae.*

## Methods

### Bacterial strains, yeast strains, and growth conditions

Restriction enzymes were purchased from New England Biolabs. PCR amplification was performed using Biolase DNA polymerase (Bioline). The yeast strain BY4742 (*MAT*α *his3*Δ*1 leu2*Δ*0 lys2*Δ*0 ura3*Δ*0*) was transformed by LiTE-Sorb method [9]. After transformation, yeast were plated on SC-Ura-Met (Sunrise Scientific) agar plates and incubated for 2 days at 30°C. Yeast were grown in liquid cultures of SC-Ura-Met at 30°C with shaking. Stock solutions of methionine and copper chloride were prepared at 1000x concentrations and added to liquid yeast cultures. The copper chelating agent BCS (bathocuproine sulfonic acid) was purchase from Sigma-Aldrich.

The bacterial strains used for protein expression were obtained from Novagen as a DE3 Competent Cell Set 1 and contained the following strains: BL21(DE3) (genotype: F^–^*ompT gal dcm lon hsdSB*(rB^-^ mB^-^) λ(DE3)); BLR(DE3) (genotype:F^-^*ompT hsdSB*(rB^-^ mB^-^) *gal dcm* (DE3) Δ(srl-recA)306::Tn10 (TetR)); HMS174(DE3) (genotype: F^-^*recA1 hsdR*(rK12^-^ mK12^+^) (DE3) (Rif R)); Turner(DE3) (genotype: F^–^*ompT hsdSB* (rB^–^ mB^–^) *gal dcm* lacY1(DE3)); and NovaBlue(DE3) (genotype: *endA1 hsdR17*(rK12^–^ mK12^+^) *supE44 thi-1 recA1 gyrA96 relA1* lac (DE3) F'[proA^+^B^+^ lacI qZDM15::Tn10] (TetR) *.*

### Isolation of plasmid DNA from *S. cerevisiae*

A 1 ml culture of yeast was pelleted and suspended in 0.5 mls of Smash and Grab buffer (20 mM Tris pH 8.0, 5 mM EDTA, 100 mM NaCl, 1% SDS, 2% Triton X-100). Cells were vortexed with acid glass beads for 1 min. The aqueous phase was isolated after 2 extractions with 0.3 mls of buffered Phenol:Chloroform:Isoamyl alcohol (25:24:1). DNA was precipitated with the addition of 3 volumes EtOH and allowed to dry. DNA was resuspended in 50 μL of TE (10 mM Tris pH 8.0, 1 mM EDTA) and incubated at 55°C for 1 hr. DNA (5 μls) was transformed in the SURE ((Stop Unwanted Rearrangement Events: e14-(McrA-) Δ(*mcrCB-hsdSMR-mrr*)*171 endA1 gyrA96 thi-1 supE44 relA1 lac recB recJ sbcC umuC::*Tn5 (Kan^r^) *uvrC* [F^′^*proAB lacIqZΔM15* Tn*10* (Tet^r^)]) cells (Strategene, Agilent, Santa Clara, CA) by electroporation. Sure cells were plated on LB-agar plates containing 100ug/ml ampicilin and grown for 18 hr at 37°C.

### Construction of the pDEP Plasmids

The yeast centromere vector pRS316 [5] was used as the basis for the pDEP vectors. A series of PCR products were produced that contained 50 bp flanking sequences of homology to the neighboring cassettes, Additional file 1: Figure S1. These homologous flanking regions were encoded in the PCR oligo primers. The DNA fragments were: the *CUP1* promoter (bp −520 to −33 of *S. cerevisiae CUP1-1* gene); a fragment containing the T7*lac* promoter and ribosomal binding site (aaggag); a sequence encoding a GST fusion protein (Glutathione-S-transferase: *Schistosoma japonicum* ) followed by a TEV (Tobacco Etch virus) protease cleavage site; and the *CYC1* 3’ untranslated region for transcription termination (bp +287 to +205 of *CYC1* gene). Vector pRS316 was digested with endonucleases NotI and KpnI. All DNA fragments were precipitated with ethanol and transformed into BY4742 yeast. After the initial pDEP plasmid was isolated from yeast, it was modified to contain a multiple cloning site from pRS306 (bp 1995–2099) by recombining a PCR amplicon containing NotI/SmaI/HindII/SalI/ and XhoI to produce pC-DEP^C^[10].

The *CUP1* promoter of the pC-DEP^C^ derivative was replaced with the methionine-regulated *MET25* promoter to produce the pM-DEP^C^ plasmid. A PCR product encoding bp −1 to −500 of MET25 gene was cotransformed with the pC-DEP^C^ plasmid digested with SacII and BamHI restriction enzymes. Recombinant plasmids were screened in yeast showing robust expression when grown in the absence methionine and showing no or modest expression in presence of 100 μM CuCl2, 200 μg/ml methionine. Both pC-DEP^C^ and pM-DEP^C^ were fully sequenced. The pC-DEP^N^ plasmid was made by first digesting pC-DEP^C^ vector with NotI and NarI. Blunt ends were created with Klenow fragment and the plasmid was ligated back together. pC-DEP-ROP was made by creating two amplicons encoding the S. pome His5 gene driven by the *TEF1* promoter and the ROP fragment. These were recombined into pC-DEP^C^ to take the place of the URA3 gene and F1 ori.

### Construction of pC-DEP^C^ containing ubiquitin-like proteins

Expression plasmids for production of GST-fused to Atg8 (residues 26–117), Esc2 (residues 389–456), Rad23 (residues 1–77), SUMO (Smt3 residues 1–101), Dsk2 (residues aa 1–77), and Hub1 (residues 1–74) were produced. PCR fragments containing the gene of interest were produced from yeast genomic DNA as template.

### Screening yeast transformants for expression of recombinant protein

Single yeast transformants were grown in 2-10 mL of yeast synthetic complete media SD-Ura-Met, in the presence of copper chloride if necessary, for 18 hr at 30°C. One OD_600_ (~ 0.5 mls) of cells were pelleted and resuspended in 100μls of volume of 0.2 M NaOH, incubated for 10 min at room temperature, and then repelleted. The cell pellet was suspended in 200μL of TWIRL buffer (8 M urea, 5% SDS, 0.4 M Tris pH 6.8) and heated to 100°C for 5 min. Samples were then immunoblotted with α-GST antibodies and positive recombinants were identified on the basis of an apparent molecular weight greater than GST (26 kDa).

### Optimized bacterial transformation and protein expression

For expression in BL21 (DE3) cells, pDEP plasmids were transformed into chemically competent cells followed by heat shock at 42°C for 30 sec and then incubated on ice for 10 min. Cells were then cultured in 20 volumes of SOC media at 37°C for 30 min and then grown overnight in 10 mls of LB + 0.25X amp containing 1% glucose. For production of recombinant proteins, bacteria were then diluted 1:100 in LB + amp media, grown for 2 hrs and induced with 0.2 mM IPTG for 2–4 hrs.

## Competing interests

The authors declare that they have no competing interest.

## Authors’ contributions

NS and CW preformed all experimental procedures and compiled data. RCP designed experiments, edited manuscript and figures. SBS composed manuscript and aided in execution of experiments. All authors read and approved the final manuscript.

## Supplementary Material

Additional file 1 **Figure S1.** Strategy for making the initial pDEP vector. Schematic of overlapping PCR products that were used in a multi-part homologous recombination event in yeast to yield the initial pDEP vector. The 3’ sequence downstream of the GST ORF of this plasmid was replaced with the multiple cloning site of pRS306 to produce the pC-DEPC plasmid. Oligonucleotides used to generate the 5 PCR products that were integrated into linearized pRS316 plasmid. Click here for file

Additional file 2 **Figure S2.** Optimization of pDEP plasmid transformation in bacterial cells. The indicated plasmids were transformed into chemically competent BL21 (DE3) cells by incubating 200 ng of plasmid with 15 μls of cells for 20 min on ice followed by 30 min recovery in SOC media. Cells were then cultured in LB media containing 50 μg/ml or 25 μg/ml ampicillin for 12 hrs. pET151 is a bacterial expression plasmid. A table showing ampicillin-resistant growth of BL21 (DE3) cells transformed with different plasmids under the indicated conditions. Note that the lower ampicillin concentration (25 μg/mL) has the largest effect increasing transformation efficiency of the pDEP plasmids. Schematic of the pC-DEP^C^-ROP plasmid. The ROP sequence and HIS5 gene replace the f1 ori and URA3 gene of the pC-DEP^C^ plasmid. The incorporation of the ROP sequence limits the plasmid copy number. Click here for file
