# Supplementary figures and images for: A set of dual promoter vectors for high throughput cloning, screening, and protein expression in eukaryotic and prokaryotic systems from a single plasmid
Source: BMC Biotechnol. 2012 Aug 23;12:54. doi: 10.1186/1472-6750-12-54 (PMC3527174; doi:10.1186/1472-6750-12-54)

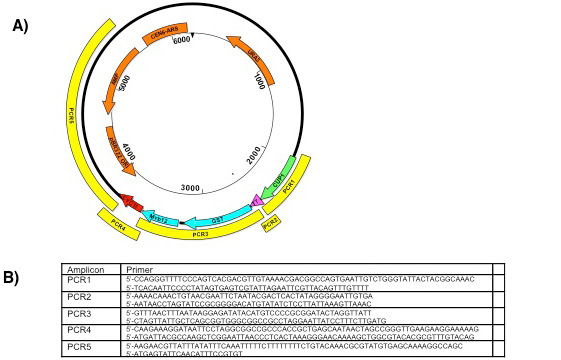

Supplement: Additional file 1 — Figure S1. Strategy for making the initial pDEP vector. Schematic of overlapping PCR products that were used in a multi-part homologous recombination event in yeast to yield the initial pDEP vector. The 3’ sequence downstream of the GST ORF of this plasmid was replaced with the multiple cloning site of pRS306 to produce the pC-DEPC plasmid. Oligonucleotides used to generate the 5 PCR products that were integrated into linearized pRS316 plasmid. [file 1472-6750-12-54-S1.jpeg]

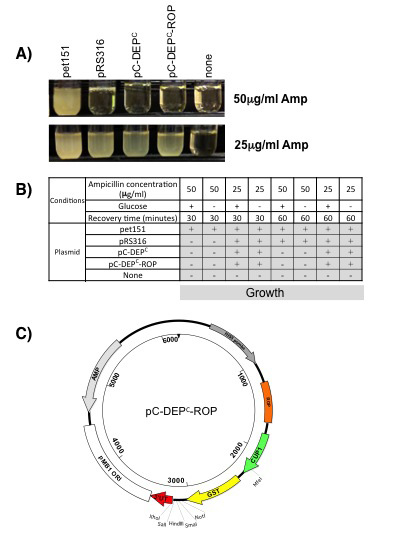

Supplement: Additional file 2 — Figure S2. Optimization of pDEP plasmid transformation in bacterial cells. The indicated plasmids were transformed into chemically competent BL21 (DE3) cells by incubating 200 ng of plasmid with 15 μls of cells for 20 min on ice followed by 30 min recovery in SOC media. Cells were then cultured in LB media containing 50 μg/ml or 25 μg/ml ampicillin for 12 hrs. pET151 is a bacterial expression plasmid. A table showing ampicillin-resistant growth of BL21 (DE3) cells transformed with different plasmids under the indicated conditions. Note that the lower ampicillin concentration (25 μg/mL) has the largest effect increasing transformation efficiency of the pDEP plasmids. Schematic of the pC-DEPC-ROP plasmid. The ROP sequence and HIS5 gene replace the f1 ori and URA3 gene of the pC-DEPC plasmid. The incorporation of the ROP sequence limits the plasmid copy number. [file 1472-6750-12-54-S2.jpeg]
